# GHSR blockade, but not reduction of peripherally circulating ghrelin via β_1_-adrenergic receptor antagonism, decreases binge-like alcohol drinking in mice

**DOI:** 10.1038/s41380-024-02713-3

**Published:** 2024-09-05

**Authors:** Rani S. Richardson, Lindsay A. Kryszak, Janaina C. M. Vendruscolo, George F. Koob, Leandro F. Vendruscolo, Lorenzo Leggio

**Affiliations:** 1 Clinical Psychoneuroendocrinology and Neuropsychopharmacology Section, Translational Addiction Medicine Branch, National Institute on Drug Abuse Intramural Research Program and National Institute on Alcohol Abuse and Alcoholism Division of Intramural Clinical and Biological Research, National Institutes of Health, Baltimore and Bethesda, MD USA; 2 Neurobiology of Addiction Section, National Institute on Drug Abuse Intramural Research Program, National Institutes of Health, Baltimore, MD USA; 3 University of North Carolina School of Medicine MD/PhD Program, University of North Carolina, Chapel Hill, NC USA; 4 Department of Cell Biology and Physiology, University of North Carolina, Chapel Hill, NC USA; 5 Stress and Addiction Neuroscience Unit, National Institute on Drug Abuse Intramural Research Program and National Institute on Alcohol Abuse and Alcoholism Division of Intramural Clinical and Biological Research, National Institutes of Health, Baltimore, MD USA; 6 National Institute on Drug Abuse Intramural Research Program Translational Analytical Core, National Institutes of Health, Baltimore, MD USA; 7 Department of Behavioral and Social Sciences, Center for Alcohol and Addiction Studies, Brown University, Providence, RI USA; 8 Medication Development Program, Molecular Targets and Medications Discovery Branch, Intramural Research Program, National Institute on Drug Abuse, Baltimore, MD USA; 9 Division of Addiction Medicine, Department of Medicine, School of Medicine, Johns Hopkins University, Baltimore, MD USA; 10 Department of Neuroscience, Georgetown University Medical Center, Washington, DC USA

**Keywords:** Neuroscience, Drug discovery

## Abstract

Alcohol use disorder (AUD) and binge drinking are highly prevalent public health issues. The stomach-derived peptide ghrelin, and its receptor, the growth hormone secretagogue receptor (GHSR), both of which are expressed in the brain and periphery, are implicated in alcohol-related outcomes. We previously found that systemic and central administration of GHSR antagonists reduced binge-like alcohol drinking, whereas a ghrelin vaccine did not. Thus, we hypothesized that central GHSR drives binge-like alcohol drinking independently of peripheral ghrelin. To investigate this hypothesis, we antagonized β_1_-adrenergic receptors (β_1_ARs), which are required for peripheral ghrelin release, and combined them with GHSR blockers. We found that both systemic β_1_AR antagonism with atenolol (peripherally restricted) and metoprolol (brain permeable) robustly decreased plasma ghrelin levels. Also, ICV administration of atenolol had no effect on peripheral endogenous ghrelin levels. However, only metoprolol, but not atenolol, decreased binge-like alcohol drinking. The β_1_AR antagonism also did not prevent the effects of the GHSR blockers JMV2959 and PF-5190457 in decreasing binge-like alcohol drinking. These results suggest that the GHSR rather than peripheral endogenous ghrelin is involved in binge-like alcohol drinking. Thus, GHSRs and β_1_ARs represent possible targets for therapeutic intervention for AUD, including the potential combination of drugs that target these two systems.

## Introduction

Alcohol use disorder (AUD) is a serious chronic disorder and a highly prevalent public health issue. Binge drinking is a harmful pattern of drinking that is an important step in the progression of AUD. Binge drinking is defined as a pattern of alcohol drinking that raises blood alcohol concentrations to 0.08 g/dl or above, which typically corresponds to consuming at least 5 drinks in males or 4 drinks in females in about 2 h [1]. Treatment of AUD and related drinking patterns (including binge drinking) is a high medical and public health priority. Yet, there are only three Food and Drug Administration (FDA)-approved pharmacological treatments for AUD; disulfiram, acamprosate and naltrexone [2]. There is a dire need for elucidating the pathophysiological mechanisms underlying AUD and the development of novel treatments. One target that is of interest for this purpose is the ghrelin system.

Ghrelin is a stomach-derived peptide secreted by P/D1cells (X/A-like cells in rodents) located primarily in the oxyntic glands of the fundus portion of the stomach [3]. Ghrelin plays key roles in regulating appetite and food intake via the growth hormone secretagogue receptor (GHSR), which is expressed in the brain and periphery [4, 5]. Liver expressed antimicrobial peptide-2 (LEAP2) is also important in this neuroendocrine system, as it acts as an endogenous GHSR inverse agonist and antagonist [6]. Ghrelin is implicated in alcohol-related outcomes in both preclinical and human studies (for reviews, see [7, 8]), including evidence that in patients with AUD, plasma ghrelin levels correlate with alcohol craving and relapse [9]. In two double-blind placebo-controlled human laboratory studies in people with AUD, intravenous ghrelin administration increased cue-induced alcohol craving in a bar-like setting [10] and intravenous alcohol self-administration using a progressive ratio schedule [11].

In mice, intracerebroventricular (ICV), intra-ventral tegmental area, and intra-dorsolateral tegmental area injections of ghrelin increased alcohol intake [12–14]. Central and peripheral ghrelin administration in male mice increased nucleus accumbens dopamine release and alcohol reward, the latter measured in a conditioned place preference (CPP) test [14]. Additionally, the pharmacological blockade or deletion of GHSR decreased alcohol consumption in mice and rats [12, 15–19]. Alcohol intake, operant alcohol self-administration, alcohol preference, alcohol-induced CPP, and alcohol-induced nucleus accumbens dopamine release have been found to be reduced in ghrelin or GHSR knock-out mice and rats [12, 14, 19, 20] and by pharmacological GHSR antagonism [12, 15–18, 21–25].

We recently found that systemic administration of GHSR antagonists, including the prototypical GHSR antagonist JMV2959 and the novel GHSR inverse agonist/antagonist PF-5190457 reduced alcohol intake in a Drinking-in-the-Dark (DID) mouse model of binge-like alcohol drinking [26]. In addition to acting as an antagonist, the inverse agonist activity of PF-5190457 blocks GHSR constitutive activity [27, 28]. Of note, we found that the ICV (i.e., central GHSR blockade) administration of JMV2959 and LEAP2 (both competitive antagonists of GHSR) reduced alcohol drinking, whereas sequestering peripherally-circulating endogenous ghrelin using a vaccine or the administration of LEAP2 systemically did not [26]. Another study showed that the neutralization of peripheral ghrelin with the oligonucleotide NOX-B11-2 did not change alcohol drinking or preference in rats tested in an intermittent access 20% alcohol 2-bottle-choice drinking paradigm, and did not change alcohol‐induced locomotor activity, alcohol-induced accumbal dopamine release, expression of alcohol conditioned place preference, or blood alcohol concentration in mice [29]. These results suggest that GHSR blockade-induced decreases in alcohol drinking may be independent from endogenous peripheral ghrelin. However, NOX-B11-2 decreased food intake in that study [29] and other studies, indicating that food intake is controlled by the peripheral ghrelin molecule [30, 31]. In another study, anti-ghrelin vaccination in mice had no effect on cocaine reward, but it blunted weight gain [32]. Taken together, these results support a role for circulating endogenous ghrelin as a physiological regulator of food and energy-related physiology, but its blockade may not be enough to influence alcohol/drug reward and seeking, for which activity at GHSR may be necessary. Indeed, ghrelin’s support of homeostatic feeding through the hypothalamus has been demonstrated to be functionally separable from its role in food reward [33, 34]. These observations led to our hypothesis that central GHSR, rather than peripheral endogenous ghrelin, controls alcohol reinforcement, reward, and alcohol-seeking behaviors. Specifically, our hypothesis was that binge-like alcohol drinking would be reduced by central GHSR antagonism by a mechanism that is independent of peripherally circulating endogenous ghrelin. Here, we investigated our hypothesis using a pharmacological approach targeting the adrenergic system.

Norepinephrine and epinephrine stimulate ghrelin secretion in vitro via β_1_ adrenergic receptors (β_1_AR) [35–37]. In vivo, the activation of β_1_AR is required for stress- and fasting-induced ghrelin release [38, 39]. The ghrelin-secreting cells contain a high density of β_1_AR, which are the only small-molecule neurotransmitter receptors enriched in ghrelin-secreting cells and are the most highly expressed of adrenergic receptors of non-odorant G-protein coupled receptors (GPCRs) in ghrelin cells [38–40]. The β_1_AR antagonist atenolol blocked cocaine self-administration, reinstatement, and cocaine-induced ghrelin release [41–43].

Even without regard to the ghrelin system, studies have linked stress-related adrenergic pathways to alcohol-related behaviors in rodents and humans. Drugs that block the βAR such as propranolol and betaxolol decreased alcohol intake in rodent models of AUD [44–60]. In male rats, propranolol (a nonselective, brain penetrant βAR antagonist) reduced operant alcohol self-administration in nondependent and alcohol-dependent rats, but with increased effect in dependent rats [46]. Propranolol suppressed the motivation to obtain alcohol in both dependent and nondependent rats in a progressive ratio test while the nonselective, peripherally-restricted βAR antagonist nadolol had no effect on responding for alcohol. In male rats, propranolol and betaxolol (β_1_AR antagonist) decreased compulsive-like alcohol drinking, whereas ICI,118,551 (a β_2_AR antagonist) had no effect [55]. In the same study, a combination of subeffective doses of propranolol and prazosin reduced compulsive-like alcohol intake. In male alcohol-preferring rats, the coadministration of prazosin and propranolol reduced alcohol drinking more effectively than either drug alone [51]. Interestingly, in a mouse model of binge drinking, lateral hypothalamus infusion of xamterol (a β_1_AR agonist) decreased drinking [60]. Recent work in male rats showed that there was no effect of propranolol on alcohol drinking when injected directly into the anterior insula or the medial prefrontal cortex [55]. Propranolol injection into the central nucleus of the amygdala (CeA) decreased alcohol intake only in alcohol-dependent rats, whereas βAR agonism disinhibited a subpopulation of CeA neurons that contributes to alcohol drinking [58]. Propranolol injected in the basolateral amygdala prevented the reinstatement of alcohol seeking in rats [59]. However, most of these studies used males only. Also, there is a paucity of information on the role of the β_1_AR and its central *versus* peripheral effects in preclinical models of binge-like alcohol drinking. Furthermore and germane to the scope of this study, there is little or no information on the crosstalk between the β_1_AR, the ghrelin system and binge-like alcohol drinking.

Cognizant of the relationship between the β_1_AR system and the ghrelin system and both being potential targets for AUD treatment, here we utilized β_1_AR antagonism-induced reduction in ghrelin secretion to elucidate the role of the peripheral ghrelin molecule in binge-like alcohol drinking in the context of pharmacological GHSR blockade. The choice of targeting selectively the β_1_AR was two-fold here. Firstly, we used it as a pharmacological probe with an established effect in depleting peripheral endogenous ghrelin [38, 41]. Secondly, the literature summarized above suggests that β_1_AR blockade may potentially have an effect on alcohol drinking.

We hypothesized that binge-like alcohol drinking is mediated by GHSRs, not by the peripherally circulating endogenous ghrelin. We utilized peripheral and central β_1_AR blockade to test the ghrelin/GHSR system’s involvement in binge-like alcohol drinking. If circulating ghrelin does not drive binge-like alcohol drinking, then the reduction of plasma ghrelin via β_1_AR antagonism should have no effect on the ability of GHSR blockers to reduce drinking. If circulating ghrelin drives alcohol drinking, then the reduction of plasma ghrelin via β_1_AR antagonism should make GHSR blockers less effective in decreasing drinking.

## Materials and methods

**Fig. 1:**
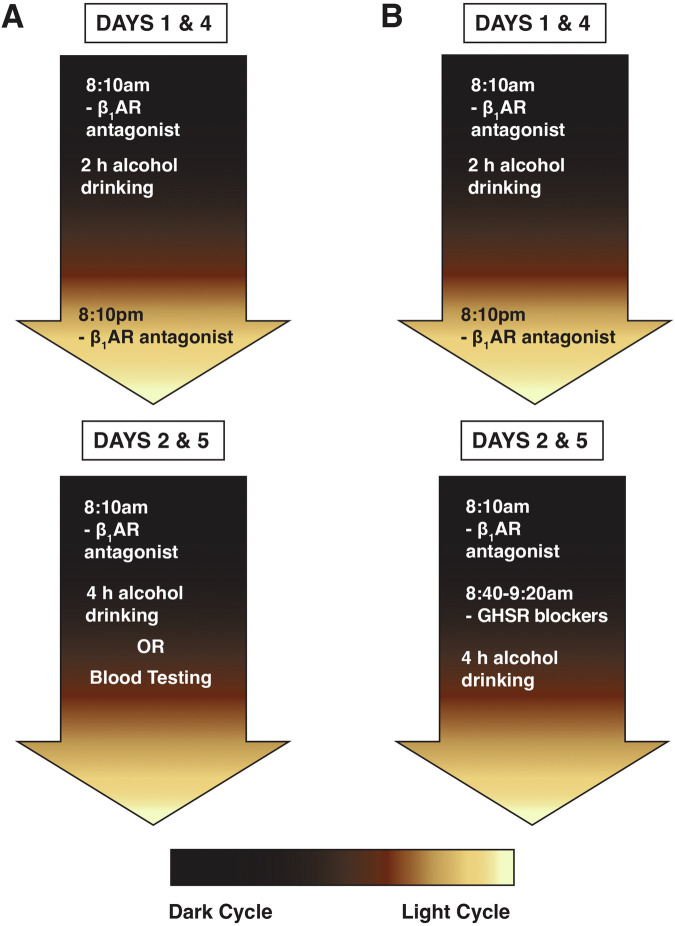
Experimental design of DID sessions. **A** Administration of β_1_AR antagonist followed by binge-like alcohol drinking or blood collection. **B** Administration of β_1_AR antagonist followed by GHSR blockers and binge-like alcohol drinking.

The experimental design and timeline are summarized in Fig. 1.

### Animals

Male (*n* = 29) and female (*n* = 30) C57BL/6 J mice were purchased from the Jackson Laboratory (Bar Harbor, ME, USA). For the systemic drug administration experiments, the mice were individually housed in standard cages and maintained in temperature- and humidity-controlled rooms. The rooms were set with a reverse 12 h/12 h light/dark cycle (lights off at 7:00 a.m.). The mice received ad libitum access to food and water, except during behavioral testing. The age of the mice was 8–13 weeks old at the start of the experiments. Behavioral testing was conducted during the dark cycle.

For the central administration experiments, a separate group of adult male (*n* = 11) and female (*n* = 11) C57BL/6 J mice were used (details below). Sample sizes were chosen based on prior published experimental methods. No randomization or blinding was used.

All procedures were performed according to the National Institutes of Health Guide for the Care and Use of Laboratory Animals and were approved by the Institutional Animal Care and Use Committee of the National Institute on Drug Abuse, Intramural Research Program.

### Drugs

Atenolol and metoprolol (Sigma Aldrich, St. Louis, MO) were dissolved in 0.9% saline (vehicle) and injected intraperitoneally (IP) at a volume of 10 ml/kg. Atenolol was administered at a dose of 20 mg/kg and metoprolol was administered at a dose of 40 mg/kg. Metoprolol is lipophilic and crosses the blood-brain barrier. Atenolol is hydrophilic and does not cross the blood-brain barrier. The doses of atenolol [35, 39] and metoprolol [61–64] and pretreatment time/schedules for administration were chosen based on prior literature. The time to peak plasma concentration (t_max_) of atenolol when given IP in mice is about 60 min, yet we could not find the t_max_ of metoprolol [65]. Drug treatment followed a between-subjects design.

In the DID experiment where atenolol or metoprolol was administered alone via IP injection, drugs were injected at 8:10 am and 8:10 pm on 2-h DID session days (*n* = 36). On 4-h session days, drug was injected at 8:10 am, which was 1.5 h prior to the beginning of DID (placing bottles in cages between 9:40 and 10 am, which is 3 h into the dark cycle). For blood collection, the mice did not receive their alcohol bottles 1.5 h after drug administration on a 4-h session day, instead cheek blood was collected for biochemical analysis.

In the DID experiment where atenolol or metoprolol were coadministered IP with a GHSR blocker. JMV2959 was dissolved in saline and administered at doses of 12 and 15 mg/kg in a Latin-square design within each β-blocker treatment group 20 min before a 4-h DID session (*n* = 36). PF-5190457 was prepared in 5% Tween 80 (v/v; Fisher Chemical, Fairlawn, NJ, USA) and saline and administered at doses of 60 mg/kg and 90 mg/kg in a Latin-square design within each β-blocker treatment group 60 min before a 4-h DID session (*n* = 34). Doses and pretreatment times for JMV2959 and PF-5190457 were chosen based on previous literature [12, 26, 66]. JMV2959 and PF-5190457 were tested sequentially in the same mice. Prior to drug testing, all mice received baseline sessions of drinking. Then, JMV2959 was administered in mice first, followed by 2 weeks of washout, then PF-5190457 administration. After JMV2959 testing, mice again received baseline drinking sessions during a washout period. During the washout period, animals were given the regular DID sessions without drug administration. During this washout period, drinking returned to similar levels as before JMV2959 treatment (7.4 g/kg before JMV2959 treatment, 7.1 g/kg during the washout). Thus, mice regained their pre-treatment baseline drinking levels prior to PF-5190457 testing. We have utilized the same experimental design in our previous study [26]. Also, we compared the drinking data from 2 h sessions that occurred within the Latin-square (i.e., the day before the treatment with JMV2959 or PF-5190457) to drinking data from sessions that occurred before the Latin-square drug testing. Average drinking during 2 h sessions that occurred before the drug testing was 3.4 g/kg, whereas average drinking during 2 h sessions that occurred during drug testing was 3.6 g/kg. Thus, we did not detect any carryover effects of drug treatment on drinking.

For the central drug administration experiment, the mice did not undergo DID or have alcohol exposure. Atenolol and JMV2959 were dissolved in 0.9% saline (vehicle) and injected into the lateral ventricle (*n* = 22). Atenolol was administered at a dose of 15 µg/µl and JMV2959 was administered at a dose of 4 µg/µl. This experiment was performed in a between-subjects design. Atenolol was injected at 8:10 am and 8:10 pm on day 1, and at 8:10 am on day 2, followed by blood collection 1.5 h later. For the JMV2959 ICV experiment, saline was injected at 8:10 am and 8:10 pm on day 1, and JMV2959 was injected at 8:10 am on day 2, followed by blood collection 1.5 h later.

### Drinking-in-the-dark (DID) test in mice

The alcohol drinking solution was prepared with 190 proof ethanol (Warner-Graham Company, Cockeysville, MD, USA) and tap water. The sweetened alcohol solution consisted of 20% alcohol (v/v), 3% glucose (w/v), and 0.1% saccharin (w/v), as previously described [26, 67, 68]. A 4-day modified protocol of DID was used [69, 70]. Mice had access to sweetened alcohol for 2 h or 4 h sessions 4 days a week. Bottles were weighed prior to and following the drinking sessions to determine intake. The difference in grams was converted to g/kg of alcohol taking into consideration the density of alcohol and the body weight of the mice. Before starting the DID schedule, water was removed overnight, and mice had access to the sweetened alcohol solution and food. Then, food and water were again given ad libitum in the home cages except during DID. During the DID sessions, water was replaced with a sweetened alcohol solution and food was removed from the cage. This occurred 3 h into the dark phase. The mice were allowed access to the drinking solutions for 2 h on day 1 and 4 h on day 2. This method was followed by 1 day off (day 3), and then the same schedule was repeated (i.e., 2 h on day 4 and 4 h on day 5). The mice were then given 2 successive days off from DID during the weekend, and DID cycles repeated weekly on weekdays. Overall, the weekly schedule consisted of 2 h drinking session on day 1; 4-h drinking session on day 2; no DID on day 3; 2-h drinking session on day 4; 4-h drinking session on day 5; no DID on days 6 and 7. Four to 6 weeks of baseline drinking were performed prior to pharmacology testing during the 4 h DID sessions. Body weights were documented weekly. To address possible spillage or leaking from the bottles, custom bottles were made in-house with double ball-bearing sippers. These sippers were tested in “sentinel” cages to assess for leaking. These metal sippers decrease the net amount of liquid leaked, thus increasing the accuracy of documented alcohol drinking.

### Locomotion

We tested the effects of atenolol and metoprolol on locomotion using drug and alcohol naïve 13-week-old mice. Each mouse was placed in a Plexiglass chamber (26 ×22.5 ×16 cm) for 10 min to habituate to the locomotion chamber. In the experiment conducted at least 24 h later, the mice received an IP administration of either vehicle, atenolol, or metoprolol in a between-subjects design. After drug administration, the mice were returned to their home cages for 90 min. They were then placed in the chamber and allowed to freely move for 10 min. Mice were then removed from the chamber and injected IP with 2 g/kg (20% w/v injection solution) alcohol in saline. Thirty min later, they were placed in the chamber again and allowed to freely move for 10 min. The total distance traveled in meters was tracked by AnyMaze Video Tracking software (Stoelting, Wood Dale, IL, USA). Immediately after this session, blood was collected from the submandibular vein to measure blood alcohol levels (BALs).

### Procedure for cannulation and infusion

For central administration experiments, adult male (*n* = 11) and female (*n* = 11) C57BL/6 J mice underwent surgery to implant cannulae that were aimed at the lateral ventricle. Mice underwent cannulation surgery at Jackson Laboratories at 11 weeks of age and were alcohol naïve. Surgery for ICV cannulae implantation utilized coordinates of 0.4 mm posterior to bregma, 1.0 mm lateral to the midline, and 2.0 mm ventral to the skull surface. Mice were habituated to the infusion procedures 2–3 times before the ICV experiments were performed. JMV 2959 and atenolol were dissolved in 0.9% saline, 0 or 4 μg/μl JMV 2959 and 0 or 15 μg/μl atenolol, with a pretreatment time of 90 min. On test days, the ICV infusions of 1 μl volume were administered over 30 s with a 10 μl Hamilton microsyringe (Hamilton Company, Reno, NV, USA). The injector remained in place for an additional 30 s to allow for diffusion and prevent reflux of the compound into the cannula. Following the drug administration, the mice were returned to their home cages, and blood samples were taken 90 min later.

### Determination of plasma ghrelin, desacyl-ghrelin and LEAP2

One and a half hours after atenolol, metoprolol, or JMV2959 administration, blood samples were collected from the submandibular vein and placed into iced EDTA-coated plastic tubes containing the protease inhibitor aprotonin (12 TIU/mL, Millipore-Sigma; Burlington, MA, USA) Following blood collection, the samples were immediately centrifuged at 4 °C, at 1600 × *g*, for 15 min. The plasma supernatant was removed and added using a ratio of to 5 µL of 1 M HCl to ensure sample stability. Plasma was stored at −80 °C until analysis. Ghrelin (acyl-ghrelin) concentrations were determined by ELISA (Bertin Pharma, Montigny-le-Bretonneux France). Desacyl-ghrelin concentrations were determined by ELISA (Bertin Pharma, Montigny-le-Bretonneux France). LEAP2 concentrations were determined by ELISA (Phoenix Pharmaceuticals, Burlingame, CA, USA). Concentrations of ghrelin, desacyl-ghrelin and LEAP2 were determined by a ClarioStar® Plus Microplate spectrophotometer (BMG Labtech, Ortenburg, Germany) and Prism version 9.3.1 software (Graphpad, San Diego, CA, USA).

### Determination of BALs

Blood samples were collected from submandibular vein using a 4 mm lancet (Medipoint, Mineola, NY, USA). Blood was taken from mice that had undergone locomotor testing and were drug and alcohol naïve prior to alcohol administration to measure BALs.

Blood was stored at 4 °C until analysis, which was performed in the same day. An ethanol calibration curve was prepared from 12.5 mg/dL to 300 mg/dL using ethanol standards (Cerilliant, Round Rock, TX, USA) in water. Briefly, 10 µL of ethanol standard or whole blood was added to 10-mL glass headspace vials (Agilent, Santa Clara, CA, USA) and sealed with a crimp cap. The vials were heated in a 70 °C 7697 A Headspace Sampler (Agilent) prior to headspace injection onto an MXT®-Volatiles 30 m, 0.28 mm ID, 1.25 µm df column (Restek, Center County, PA, USA) using helium as the carrier gas. The 8890 gas-chromatography system column oven (Agilent) was heated to 40 °C for an isocratic 6 min run paired with a 5977B gas-chromatograph/mass selective detector (Agilent).

### Statistical analysis

Because we did not find sex differences in the effects of JMV2959 and PF-5190457 in reducing binge-like alcohol drinking in mice in our previous study [26], in the present study, we used mixed cohorts of males and females and analyzed the data without a Sex factor. The drinking data were examined using one-way, two-way, or 3-way repeated-measures analysis of variance (ANOVA) or a mixed model analysis (two-sided). For the coadministration experiments where GHSR blocker and β_1_AR blocker were co-administered in the same mouse, GHSR blocker Treatment was used as within-subjects factor and β_1_AR blocker Treatment was used as between-subjects factor. We conducted *post hoc* comparisons using the Dunnett’s test following significant GHSR blocker Treatment effects, β_1_AR blocker Treatment effects, and GHSR blocker Treatment x β_1_AR blocker Treatment interactions. For analysis of ghrelin, des-acyl ghrelin and LEAP2 levels, one-way analysis of variance (ANOVA) was used followed by *post hoc* comparisons using the Dunnett’s test. For analysis of spontaneous locomotion data, a two-way ANOVA was performed. For analysis of BALs, a one-way ANOVA was performed. All data are presented as the mean and standard error of the mean (SEM), and individual data points are shown where appropriate. Grubb’s test was used to identify significant outliers. Statistical significance was considered at *p* < 0.05 for all tests. Analyses including the Sex factor are reported in Supplemental Table 1–3. All analyses were performed using Prism 9.3.1 software (GraphPad, San Diego, CA, USA).

## Results

### Effects of β_1_AR blockade on plasma hormone levels

**Fig. 2:**
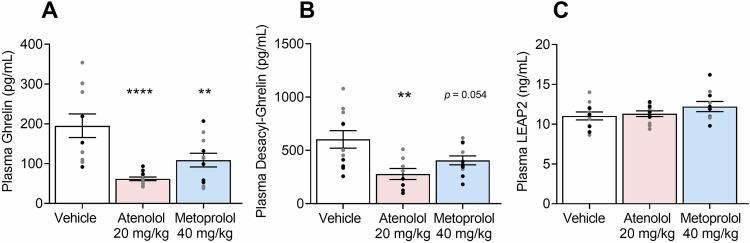
Effects of β_1_AR blockade on plasma peptide levels. **A** Atenolol and metoprolol reduced ghrelin levels. **B** Atenolol reduced desacyl-ghrelin levels, but metoprolol did not. **C** Atenolol or metoprolol had no effect on LEAP2 levels. For each drug, *n* = 12 (6 males, 6 females). Individual values for males () and females () are shown along with mean ± SEM. *p < 0.05, **p < 0.01, ****p < 0.0001, difference from vehicle.

There was a β_1_AR-antagonist Treatment effect on plasma ghrelin levels (*F*_2,31_ = 12.25, *p* = 0.0001; Fig. 2). Compared to vehicle, both atenolol (*p* < 0.0001) and metoprolol (*p* = 0.0076), administered IP, decreased plasma ghrelin levels. There was also a β_1_AR antagonist Treatment effect on desacyl-ghrelin levels (*F*_2,27_ = 6.392, *p* = 0.0053), with atenolol (*p* = 0.0033) but not metoprolol (*p* = 0.0537) decreasing des-acyl-ghrelin levels. There was no effect of atenolol or metoprolol on plasma LEAP2 levels (*F*_2,29_ = 1.433, *p* = 0.2551).

### Effects of β_1_AR blockade on binge-like alcohol drinking, locomotion, and BALs

**Fig. 3:**
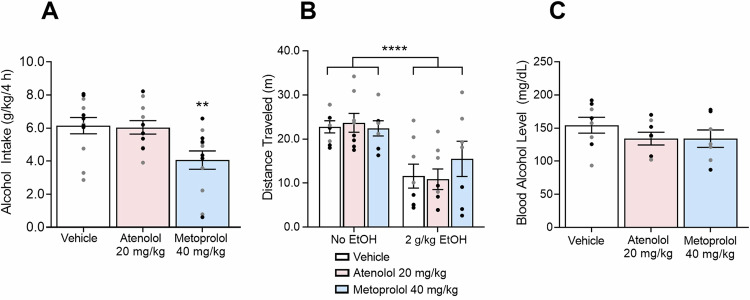
Effect of β_1_AR blockade on binge-like alcohol drinking, locomotion, and BALs. **A** Metoprolol, but not atenolol, significantly decreased alcohol (20% v/v) intake (g/kg of bodyweight) in mice. For each drug, *n* = 12 (6 males, 6 females). **B** Atenolol and metoprolol had no effect on locomotion (distance traveled) when administered alone or with a 2 g/kg bolus alcohol injection. Alcohol decreased locomotion. **C** Atenolol and metoprolol had no effect on BALs. For each drug, *n* = 7–8 (11 males, 12 females total). Individual values for males () and females () are shown along with mean ± SEM. **p < 0.01, ****p < 0.0001, difference from vehicle.

Figure 3 shows the effects of atenolol and metoprolol (IP) on binge-like alcohol drinking, locomotion, and BALs. For alcohol drinking, there was a β_1_AR antagonist Treatment effect: (*F*_4,32_ = 5.699, *p* = 0.0076). Atenolol (does not cross the blood-brain barrier) had no effect on binge-like alcohol drinking (*p* = 0.9848), whereas metoprolol (crosses the blood-brain barrier) decreased binge-like alcohol drinking compared with vehicle-treated mice (*p* = 0.0096). To investigate whether β_1_AR antagonism has effects on locomotion, we administered the β_1_AR antagonists alone and in combination with 2 g/kg alcohol. Without alcohol, the β_1_AR blocker treatments did not affect locomotion (*F*_2,20_ = 0.2207, *p* = 0.8039). Following alcohol administration, there was an effect of alcohol administration on locomotion (*F*_1,20_ = 44.21, *p* < 0.0001), indicating a decrease in locomotion following alcohol treatment. However, β_1_AR blocker Treatment did not affect locomotion (β_1_AR blocker Treatment: *F*_2,20_ = 0.2207, *p* = 0.8039; β_1_AR blocker Treatment x Alcohol Treatment interaction: *F*_2,20_ = 1.237, *p* = 0.3114). The β_1_AR blocker treatment did not affect BALs (*F*_2,20_ = 1.053, *p* = 0.3675).

### Effect of the coadministration of JMV2959 and β_1_AR blockers on binge-like alcohol drinking

**Fig. 4:**
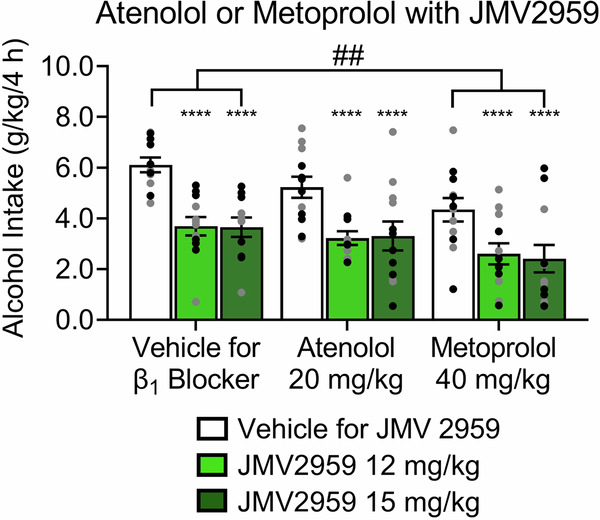
Effect of the coadministration of the GHSR blocker JMV2959 and β_1_AR blockers on binge-like alcohol drinking. JMV2959 decreased alcohol (20% v/v) intake (g/kg of bodyweight) in mice. Metoprolol decreased alcohol intake, whereas atenolol had no effect on alcohol intake. For each drug, *n* = 12 (6 males, 6 females). Individual values for males () and females () are shown along with mean ± SEM. ^##^*p* < 0.01, difference between vehicle and metoprolol; ****p < 0.0001, difference between vehicle and JMV2959.

To investigate whether the β_1_AR-blocker-induced reduction of plasma ghrelin levels (Fig. 2) would alter the ability of the GHSR antagonist JMV2959 to reduce binge-like alcohol drinking, we co-administered JMV2959 with atenolol or metoprolol via IP administration. Both doses of JMV 2959 (*p* < 0.0001) reduced binge-like alcohol drinking (JMV2959 Treatment effect: *F*_2,64_ = 30.25, *p* < 0.0001; Fig. 4). Moreover, β_1_AR antagonist Treatment (*F*_2,33_ = 5.291, *p* = 0.0102) changed alcohol drinking. Metoprolol decreased binge-like alcohol drinking (*p* = 0.0053), whereas atenolol had no effect (*p* = 0.3238). The JMV2959 Treatment x β_1_AR antagonist Treatment interaction was not statistically significant (*F*_4,64_ = 0.2852, *p* = 0.8866).

### Effect of the coadministration of PF-5190457 and β_1_AR blockers on binge-like alcohol drinking

**Fig. 5:**
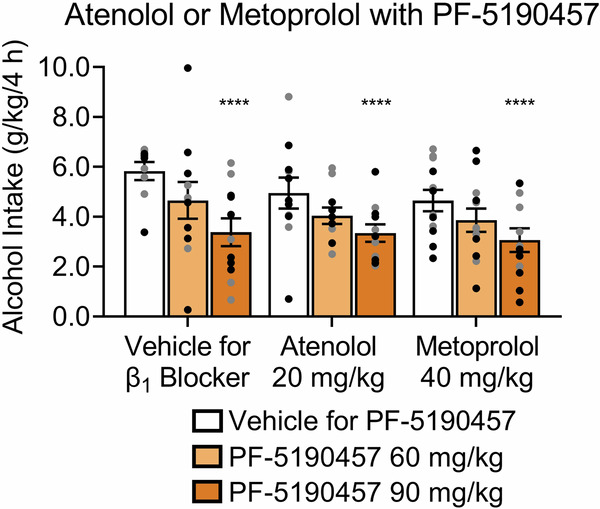
Effect of the coadministration of the GHSR blocker PF-5190457 and β_1_AR blockers on binge-like alcohol drinking. PF-5190457 decreased alcohol (20% w/v) intake (g/kg of bodyweight) in mice. For vehicle and atenolol groups, *n* = 11 (6 males, 5 females) and for metoprolol *n* = 12 (6 males, 6 females). Individual values for males () and females () are shown along with mean ± SEM. ****p < 0.0001, difference from vehicle.

We also co-administered PF-5190457 with atenolol or metoprolol in the same mice via IP administration. PF-5190457 at 90 mg/kg (*p* < 0.0001) reduced binge-like alcohol drinking (PF-5190457 Treatment effect: *F*_2,62_ = 12.60, *p* < 0.0001; Fig. 5). β﻿_1_AR antagonist Treatment (*F*_2,31_ = 1.53, *p* = 0.2325) had no effect on alcohol intake. The PF-5190457 Treatment x β_1_AR antagonist Treatment interaction was not statistically significant (*F*_4,62_ = 0.3072, *p* = 0.8271).

### Effect of central administration of atenolol and JMV2959 on plasma ghrelin levels

**Fig. 6:**
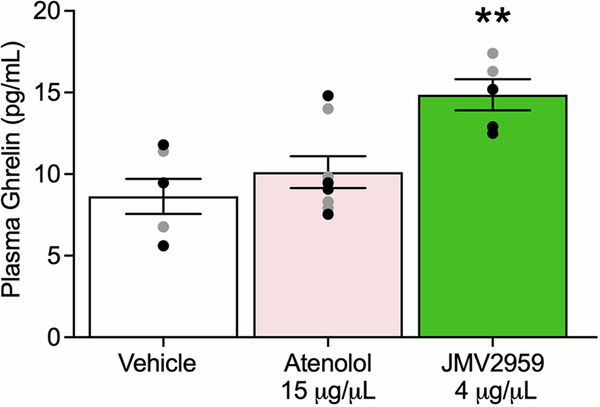
Effects of central β_1_AR blockade and GHSR blockade on plasma peptide levels. Atenolol had no effect on plasma ghrelin levels, but JMV2959 increased plasma ghrelin levels. For each drug, *n* = 7–8 (3–4 males, 3–4 females). Individual values for males () and females () are shown along with mean ± SEM. **p < 0.01.

We investigated whether the antagonism of central β_1_AR or GHSR affects plasma ghrelin levels. In a between-subjects design experiment, we administered atenolol or JMV2959 via ICV route and subsequently measured plasma ghrelin levels. There was a Treatment effect (F_2,16_ = 8.62, *p* = 0.0029). Post hoc comparisons indicated that atenolol had no effect on peripheral endogenous ghrelin levels compared to vehicle, but JMV2959 increased peripheral endogenous ghrelin levels (*p* = 0.0020) (Fig. 6).

## Discussion

We found that the systemic administration of both the brain-penetrant β_1_AR antagonist metoprolol and the peripherally restricted β_1_AR antagonist atenolol substantially decreased plasma ghrelin levels. However, this marked decrease in endogenous peripheral ghrelin levels did not prevent the GHSR antagonists JMV2959 and PF-5190457 from reducing binge-like alcohol drinking. These findings suggest that the ability of GHSR blockade to reduce binge-like alcohol drinking is independent from peripherally circulating endogenous ghrelin. These findings also indicate that peripheral endogenous ghrelin may not drive binge-like alcohol drinking. Furthermore, we found in alcohol-naive mice that the central administration of atenolol did not alter endogenous peripheral ghrelin levels, suggesting that systemically-administered β_1_AR antagonists depleted ghrelin levels via peripheral β_1_AR receptors. Moreover, we performed additional analyses on our DID data that incorporated Sex as a factor and we did not observe treatment differences between males and females (Supplemental Table S1-S3).

Although both metoprolol and atenolol reduced peripheral endogenous ghrelin levels, only metoprolol decreased binge-like alcohol drinking. Neither atenolol nor metoprolol affected locomotion or BALs. Together, these results show that central but not peripheral β_1_AR antagonism reduces binge-like alcohol drinking, hence suggesting that binge-like alcohol drinking may be triggered by activation of the central autonomic adrenergic system.

LEAP2 is a recently discovered endogenous antagonist of the ghrelin system that is primarily secreted by the liver [6]. Circulating LEAP2 and ghrelin inversely correlate with body mass, indicating that they act in tandem in response to energy demand. LEAP2 antagonizes the actions of ghrelin, and both have similar potency and affinity for GHSR [71]. LEAP2 seems to be sensitive to body weight and feeding status and may be controlled in a manner opposite to that of plasma ghrelin. In our study, there was an effect of β_1_AR antagonism on plasma ghrelin but not LEAP2. The lack of LEAP2 response suggests that the secretion of these two hormones can be dissociated under certain circumstances. Given that herein β_1_AR antagonists were administered to *ad libitum* fed mice, further experiments should evaluate the role of atenolol and metoprolol in fasted mice.

Once secreted into the circulation, ghrelin can be deacylated by plasma enzymes to form desacyl-ghrelin [72]. Desacyl-ghrelin is the predominant form of ghrelin present in the circulation. However, its functions, if any, are not well understood, nor has its putative receptor been identified [73–75]. Albeit the role of desacyl-ghrelin is controversial, it has been shown in some studies that desacyl-ghrelin mediates food intake and metabolism with effects that may be antagonistic to the effects of ghrelin [74]. Desacyl-ghrelin recently has been purported to exert its physiological roles through a GHSR-independent mechanism [73–75]. In the present study, atenolol decreased desacyl-ghrelin levels at a statistically significant level, whereas metoprolol decreased it at a trend level. This could potentially be explained by atenolol being more effective in decreasing plasma ghrelin levels than metoprolol, thus less ghrelin available to be deacylated. This is consistent with the finding that the calculated des-acyl-ghrelin/ghrelin molar ratios for atenolol and metoprolol were similar.

We found that the GHSR blockers JMV2959 and PF-5190457 reduce binge-like alcohol drinking even when peripheral endogenous ghrelin levels were depleted via atenolol and metoprolol. It is unclear how GHSR blockade remains effective in reducing alcohol drinking when ghrelin has been significantly lowered in the periphery. A possible explanation would be that ghrelin is produced centrally, and this is driving alcohol drinking. Indeed, it has been speculated that ghrelin is synthesized centrally [76], yet the role and physiological efficacy of centrally-produced ghrelin remains a controversial topic in the field. Another explanation is that even in the presence of atenolol or metoprolol, which robustly diminished plasma ghrelin levels, the peptide is not eliminated. Albeit in small quantities, some ghrelin may remain in the periphery to mediate drinking, or it may access the brain to mediate drinking [77–80]. Yet, this is unlikely given dose-dependent effects that we observed with GHSR blockers [26]. Future studies should further investigate the role of endogenous central nervous system ghrelin itself in AUD, e.g., experiments in ghrelin peptide knockout mice. Another possible explanation lies with the dimerization of GHSR. GHSR can form heterodimers with receptors for other neurotransmitters, including oxytocin, dopamine, and serotonin [27, 81]. Thus, it is possible that even with peripheral ghrelin depletion, GHSR can still modulate binge-like alcohol drinking via interactions with other receptors that are known to be involved in drug reward and addiction. Future studies are necessary to verify whether GHSR heterodimerization is involved in binge-like alcohol drinking.

It, also, is important to consider our results in the context of prior literature. The antagonism of peripheral β_1_AR by atenolol potently attenuated the elevation in circulating ghrelin induced by cocaine [41, 42]. Atenolol inhibited cocaine self-administration and cocaine-induced reinstatement. Cocaine differs from alcohol in that while cocaine increases plasma ghrelin, alcohol decreases plasma ghrelin (for review, see [7]). It is possible that one reason atenolol had no effect on binge-like alcohol drinking is that, unlike cocaine, alcohol does not stimulate ghrelin release. Thus, ghrelin levels would be theoretically low in response to alcohol even without atenolol present, which further supports our interpretation of a decrease in alcohol drinking independent from peripheral endogenous ghrelin.

In the present study, the β_1_AR antagonists showed no effect on spontaneous locomotion. One point of consideration is the question surrounding the sedative effects of GHSR blockade. For JMV2959, there are a few cases in the literature where there is an effect of JMV2959 on baseline locomotion, albeit the effect is mild and transient. [82, 83] Also, literature has shown that even at the dose of 6 mg/kg, JMV2959 does not affect locomotion per se [12, 22, 84–86]. JMV2959 alone usually has no effects on animals’ locomotor activity. Additionally, in our previous study, JMV2959 12 mg/kg and PF-5190457 (90 mg/kg) blocked intake of sweetened and unsweetened alcohol solutions [26]. However, these doses had no effect on the intake of a non-alcohol containing sweet solution, indicating that the effects of JMV2959 and PF-5190457 on alcohol intake were likely related to the reinforcing effects of alcohol rather than any potential effects on locomotion. Consistent with the preclinical work, a Phase 1b human study with people who were heavy alcohol drinkers did not find an effect of PF-5190457 on sedation, either alone or combined with alcohol [66].

One potential anatomical point of convergence between adrenergic and ghrelin systems is the hypothalamus. In rodents, the hypothalamus has been implicated in modulating alcohol consumption and seeking [87–93]. In general, stimulation of adrenergic receptors in the rat hypothalamus affects eating and water drinking behaviors [94–99]. For example, stimulation of hypothalamic αAR elicited feeding behaviors and suppressed water drinking [96–98]. These effects seem to be mediated by the ventromedial hypothalamus. In contrast, stimulation of βARs suppressed eating and stimulated water drinking. These systems are thought to be connected by reciprocal circuits that act antagonistically to regulate hunger and thirst [100]. It is possible that the mechanism by which antagonism of central β_1_AR decreased alcohol intake in our study involves antagonism of hypothalamic β_1_AR. In addition, the hypothalamus is an important site of ghrelin system action. GHSR is highly expressed the ventromedial and arcuate nucleus of the hypothalamus, but is not expressed in the lateral hypothalamus. This may explain why, in prior studies, ghrelin had no effect on alcohol intake when administered into the lateral hypothalamus, but increased alcohol intake when administered to other brain areas [12, 101]. The interactions between hypothalamic βAR signaling and ghrelin signaling should be interrogated in future studies.

Another point of interest is the HPA axis. The largest population of noradrenergic neurons is in the bilateral locus coeruleus (LC). The adrenergic system and the HPA axis have a bidirectional relationship. For example, CRF afferents from areas such as the CeA innervate the LC and influence its activity. The LC’s outputs regulate CRF containing neurons in the CeA, and activate the CRF neurons in the PVN [102]. Also, alcohol itself affects adrenergic and CRF signaling; for example, the LC is activated following voluntary binge-like alcohol drinking [60, 103]. One study showed that LC lesions blocked the effects of centrally injected alcohol on stimulation of ACTH secretion [102]. It is possible that β_1_AR antagonism operates via CRF signaling to affect binge-like alcohol drinking.

Furthermore, the ghrelin system plays a key role signaling stress to the brain [104]. Ghrelin stimulates the HPA axis, and glucocorticoids stimulate ghrelin release. Notably, intact ghrelin signaling appears to be required for full activation of the HPA axis under certain stressful conditions [105, 106]. Ghrelin activates hypothalamic CRF neurons (cFos in CRF-producing neurons and CRF gene expression), but this effect is independent of GHSR, as CRF neurons in the PVN do not express GHSR. This effect seems to be mediated by changes in local GABAergic tone [107]. These data suggest that the neuronal circuits mediating ghrelin’s orexigenic action compared with its role as a stress signal are anatomically dissociated. This is not surprising given that ghrelin’s effects on food reward and homeostatic eating are also dissociated [33]. It is possible that ghrelin-controlled activity of these dissociated neuronal circuits depends on the levels of plasma ghrelin present under different conditions; given that small increases in circulating ghrelin mainly impact the arcuate nucleus whereas higher increases in circulating ghrelin can have an effect on other brain areas such as the PVN [107, 108]. An intact HPA axis appears to serve as an inhibitory feedback system, as both central CRF agonists and systemic glucocorticoid administration significantly inhibit ghrelin secretion, whereas adrenalectomy-induced elimination of corticoids potentiates fasting-induced ghrelin elevation, an effect that can be normalized by glucocorticoid replacement. Also, importantly, the site of action of norepinephrine in addiction likely involves the extended amygdala rather than the locus coeruleus [109]. Glucocorticoids attenuate CRF activity in the hypothalamus but stimulate activity in the extended amygdala [110]. In fact, a critical portion of the brain’s norepinephrine is derived from the bed nucleus of the stria terminalis. This portion comes from the ventral noradrenergic bundle which derives from sub-coerulear norepinephrine nuclei in the brain stem, rather than coming from the locus coeruleus (the dorsal noradrenergic bundle) [109]. Additionally, in previous work, atenolol decreased plasma ghrelin not only in fasted animals, but also in ad libitum fed animals [39] and only fasted animals manifest hypoglycemia in response to β_1_AR antagonist-mediated blockade of ghrelin release [39]. In the present study, mice were fed ad libitum, hence future studies should expand the present findings to mice that are food restricted.

Finally, we tested the effects of central JMV2959 administration on peripheral ghrelin levels. We administered JMV2959 ICV and found that the central administration of JMV2959 increased peripheral ghrelin levels, suggesting a role of central GHSR in negative feedback. The results could possibly be due to a feedback mechanism that involves increased peripheral ghrelin secretion in response to GHSR blockade in an effort to maintain homeostasis (i.e. blood glucose levels). Overall, the finding that ICV JMV2959 can increase peripheral ghrelin at an identical dose that leads to decreased drinking [26] may support the notion that binge-like alcohol drinking occurs independently of peripheral ghrelin levels. Furthermore, given that central but not peripheral β_1_AR antagonism reduced drinking, while peripheral but not central β_1_AR antagonism depleted peripheral endogenous ghrelin levels, the results further suggest that the effect of β_1_AR antagonism on binge-like alcohol drinking is independent from peripherally circulating endogenous ghrelin levels.

In summary, systemic but not central β_1_AR antagonism decreased plasma ghrelin levels but did not prevent GHSR blockade in reducing binge-like alcohol drinking. Central but not peripheral β_1_AR antagonism decreased binge-like alcohol drinking. These findings suggest that the effects of central GHSR blockade and central β_1_AR antagonism in reducing alcohol drinking are independent from peripherally circulating endogenous ghrelin in the modulation of binge-like alcohol drinking.

## Supplementary information

Supplementary Material

## Data Availability

Relevant data generated and analyzed during this study are included in this article and its supplementary information.
