# Supplementary material for: GHSR blockade, but not reduction of peripherally circulating ghrelin via β1-adrenergic receptor antagonism, decreases binge-like alcohol drinking in mice
Source: Mol Psychiatry. 2024 Sep 5;30(3):1047–56. doi: 10.1038/s41380-024-02713-3 (PMC11835741; doi:10.1038/s41380-024-02713-3)
Supplement: Supplementary file 1 — Supplementary Material [file 41380_2024_2713_MOESM1_ESM.docx]

**SUPPLEMENTARY INFORMATION**

**GHSR blockade, but not reduction of peripherally circulating ghrelin via β1-adrenergic receptor antagonism, decreases binge-like alcohol drinking in mice**

Rani S. Richardson, Lindsay A. Kryszak, Janaina C. M. Vendruscolo, George F. Koob, Leandro F. Vendruscolo*, Lorenzo Leggio*

*Co-Senior Authors

| **Table S1**. Mixed methods analysis of data on blood hormones, binge-like alcohol drinking and locomotion in response to β_1_AR blockers with Sex and β_1_AR blocker Treatment as factors. | | | |
| --- | --- | --- | --- |
| **Measured Outcome** | **Sex** | **β_1_AR Blocker Treatment** | **Sex × Treatment interaction** |
| **Ghrelin** | *F*_1,28_ = 0.08750, *p* = 0.7696 | *F*_2,28_ = 11.13, *p* = 0.0003 | *F*_2,28_ = 0.5237, *p* = 0.5980 |
| **Desacyl-ghrelin** | *F*_1,24_ = 10.56, *p* = 0.0034, F > M | *F*_2,24_ = 8.808, *p* = 0.0014 | *F*_2,24_ = 1.217, *p* = 0.3137 |
| **LEAP2** | *F*_1,26_ = 0.06565, *p* = 0.7998 | *F*_2,26_ = 1.534, *p* = 0.2347 | *F*_2,26_ = 1.577, *p* = 0.2 |
| **^Drinking (β_1_AR blocker)** | *F*_1,29_ = 1.483, *p* = 0.2331 | *F*_2,29_ = 5.718, *p* = 0.0081 | *F*_2,29_ = 0.7332, *p* = 0.4891 |
| **Locomotion no alcohol** | *F*_1,23_ = 24.35, *p* < 0.0001, F > M | *F*_2,23_ = 0.1900, *p* = 0.8282 | *F*_2,23_ = 0.6706, *p* = 0.5211 |
| **Locomotion with alcohol** | *F*_1,17_ = 47.80, *p* < 0.0001, F > M | *F*_2,17_ = 1.077, *p* = 0.3628 | *F*_2,17_ = 1.535, *p* = 0.2439 |
| **Blood alcohol levels** | *F*_1,20_ = 0.2065, *p* =0.6544 | *F*_2,20_ = 0.3033, *p* = 0.7417 | *F*_2,20_ = 0.007102, *p* = 0.9929 |

^Drinking (β_1_AR blocker) refers to mice administered intraperitoneal atenolol or metoprolol prior to drinking-in-the-dark (DID) session

LEAP2: liver-expressed antimicrobial peptide-2, EtOH: alcohol, BALs: blood alcohol levels, F: female, M: male, β_1_AR: beta-1 adrenergic receptor

Note: The statistical values in Supplemental Tables 1-3 show results from analyses that incorporated Sex as a factor, whereas the analyses in the main manuscript did not include Sex as a factor.

**Table S2**. Three-way ANOVA for the JMV2959-β_1_AR co-administration experiment with Sex, JMV2959 treatment, and β_1_AR blocker Treatment as factors.

| **Sex** | *F*_1,30_ = 0.4410, *p* = 0.5117 |
| --- | --- |
| **β_1_AR blocker Treatment^#^** | *F*_2,30_ = 5.7311, *p* = 0.0078 |
| **JMV2959 Treatment^##^** | *F*_2,60_ = 30.9660 *p* = 0.0000 |
| **Sex × β_1_AR blocker Treatment interaction** | *F*_2,30_ = 1.9100, *p* = 0.1652 |
| **Sex × JMV2959 Treatment interaction** | *F*_2,60_ = 0.0194 *p* = 0.9808 |
| **JMV2959 Treatment × β_1_AR blocker Treatment interaction** | *F*_4,60_ = 0.2612 *p* = 0.9017 |
| **JMV2959 Treatment × Sex × β_1_AR blocker Treatment interaction** | *F*_4,60_ = 1.3556 *p* = 0.2601 |

^#^Post hoc comparisons indicated that metoprolol (*p* = 0.004) but not atenolol (*p* = 0.298) decreased alcohol intake

^##^Post hoc comparisons indicated that both doses of JMV2959 had an effect (*p* < 0.0001)

**Table S3**. 3-way ANOVA data for the PF-5190457-β_1_AR co-administration experiment with Sex, PF-5190457 administration, and β_1_AR blocker Treatment as factors.

| **Sex** | *F*_1,28_ = 1.4700, *p* = 0.2355 |
| --- | --- |
| **Dose of β_1_AR blocker Treatment** | *F*_2,28_ = 1.6337, *p* = 0.2133 |
| **Dose of PF-5190457*** | *F*_2,56_ = 12.4870 *p* = 0.0000 |
| **Sex × β_1_AR blocker Treatment interaction** | *F*_2,28_ = 0.6631, *p* = 0.5232 |
| **Sex × PF-5190457 interaction** | *F*_2,56_ = 0.1123 *p* = 0.8940 |
| **PF-5190457 × β_1_AR blocker Treatment interaction** | *F*_4,56_ = 0.3097 *p* = 0.8703 |
| **PF-5190457 × Sex × β_1_AR blocker Treatment interaction** | *F*_4,56_ = 1.4172 *p* = 0.2402 |

*Post hoc comparisons indicated that both 60 mg/kg (*p* = 0.0283) and 90 mg/kg (*p* < 0.0001) of PF-5190457 decreased drinking
